# 

**Published:** 1845-11

**Authors:** 


CONTENTS:

Diseases of the Summer and Autumn of 1845. - - - 113

BIBLIOGRAPHICAL NOTICES.

The Principles and Practice of Obstetric Medicine and Surgery By FRANCIS H. RAMSBOTHAM, M.D. - - - - - 115
A Dictionary of Terms used in Medicine and the Collateral Sciences, By RICHAR HOBLYN, M.D. - - - - - 116
The Half-Yearly Abstract of the Medical Sciences, By W. H. RANKING, M. D. - - 116

MEDICAL INTELLIGENCE.

To the Medical Public. - 117

PRACTICAL MEDICINE, &C.

Brodie's Surgical Lectures. - 117
Gout and Rheumatism—their Pathology and Treatment. - 122
Case of Acites, cured by Injection of a Stimulating Fluid into the Peritoneal Cavity. - - 125
Delirium Tremens. - - 126
Extreme Mercurial Salivation. 127

